# A phase 1b open-label dose-finding study of ustekinumab in young adults with type 1 diabetes

**DOI:** 10.1093/immadv/ltab022

**Published:** 2021-11-13

**Authors:** Ashish K Marwaha, Samuel Chow, Anne M Pesenacker, Laura Cook, Annika Sun, S Alice Long, Jennie H M Yang, Kirsten A Ward-Hartstonge, Evangelia Williams, Clara Domingo-Vila, Khalif Halani, Kristina M Harris, Timothy I M Tree, Megan K Levings, Thomas Elliott, Rusung Tan, Jan P Dutz

**Affiliations:** 1 Department of Medical Genetics, University of Calgary, Cumming School of Medicine, Calgary, Alberta, Canada; 2 Department of Dermatology and Skin Science, University of British Columbia, Vancouver, British Columbia, Canada; 3 BC Children’s Hospital Research Institute, Vancouver, British Columbia, Canada; 4 Department of Surgery, University of British Columbia, Vancouver, British Columbia, Canada; 5 Department of Medicine, University of British Columbia, Vancouver, British Columbia, Canada; 6 Benaroya Research Institute at Virginia Mason, Translational Research Program, Seattle, WA, USA; 7 Department of Immunobiology, King’s College London, London, UK; 8 Emmes Canada, Burnaby, British Columbia, Canada; 9 Immune Tolerance Network, Bethesda, MD, USA; 10 BCDiabetes, Vancouver, British Columbia, Canada; 11 Department of Pathology, Sidra Medicine and Weill Cornell Medicine, Doha, Qatar; 12 Department of Pathology and Laboratory Medicine, University of British Columbia, Vancouver, British Columbia, Canada

**Keywords:** ustekinumab, type 1 diabetes, clinical trial, immunomodulatory

## Abstract

**Objectives:**

We assessed the safety of ustekinumab (a monoclonal antibody used in psoriasis to target the IL-12 and IL-23 pathways) in a small cohort of recent-onset (<100 days of diagnosis) adults with type 1 diabetes (T1D) by conducting a pilot open-label dose-finding and mechanistic study (NCT02117765) at the University of British Columbia.

**Methods:**

We sequentially enrolled 20 participants into four subcutaneous dosing cohorts: (i) 45 mg loading weeks 0/4/16, (ii) 45 mg maintenance weeks 0/4/16/28/40, (iii) 90 mg loading weeks 0/4/16, and (iv) 90 mg maintenance weeks 0/4/16/28/40. The primary endpoint was safety as assessed by an independent data and safety monitoring board (DSMB) but we also measured mixed meal tolerance test C-peptide, insulin use/kg, and HbA1c. Immunophenotyping was performed to assess immune cell subsets and islet antigen-specific T cell responses.

**Results:**

Although several adverse events were reported, only two (bacterial vaginosis and hallucinations) were thought to be possibly related to drug administration by the study investigators. At 1 year, the 90 mg maintenance dosing cohort had the smallest mean decline in C-peptide area under the curve (AUC) (0.1 pmol/ml). Immunophenotyping showed that ustekinumab reduced the percentage of circulating Th17, Th1, and Th17.1 cells and proinsulin-specific T cells that secreted IFN-γ and IL-17A.

**Conclusion:**

Ustekinumab was deemed safe to progress to efficacy studies by the DSMB at doses used to treat psoriasis in adults with T1D. A 90 mg maintenance dosing schedule reduced proinsulin-specific IFN-γ and IL-17A-producing T cells. Further studies are warranted to determine if ustekinumab can prevent C-peptide AUC decline and induce a clinical response.

## Graphical Abstract

**Figure:**
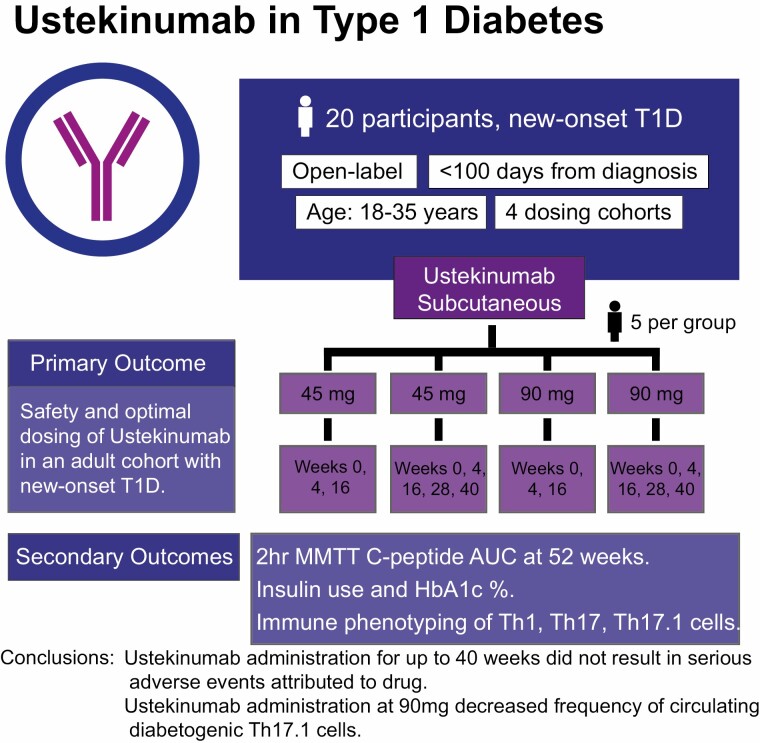

## Introduction

Type 1 diabetes (T1D) is an autoimmune disease that arises from the T cell-mediated destruction of pancreatic β-cells. Data generated from people (individuals) with recent-onset T1D [1], indicate that functional insulin-secreting β-cells are present at time of disease presentation. Thus, long-term interruption of T cell-mediated, autoimmune β-cell destruction at the time of clinical T1D presentation could preserve sufficient β-cells to maintain insulin secretion.

T1D pathogenesis involves defects in immune tolerance, particularly in CD4^+^CD25^+^FOXP3^+^ regulatory T cells (Tregs), permitting expansion of autoreactive CD4^+^ and CD8^+^ T cells [2], which destroy insulin-producing β-cells. Peripheral blood mononuclear cells (PBMC) from children with recent-onset T1D [3] have a higher proportion of CD4^+^ and CD8^+^ T cells that secrete IL-17A (known as Th17 and Tc17 cells, respectively) [4]. Dysregulated IL-17A production acts in concert with IFN-γ, an inflammatory and cytotoxic cytokine produced by Th1 cells. A subset of Th17 cells, termed Th17.1 cells, simultaneously produces IFN-γ, and IL-17A, and are defined as ‘non-classical’ Th1 cells [5]. Although there is conflicting evidence regarding the role of Th17 cells in the non-obese diabetic (NOD) mouse model, the Th1 pathway is implicated in disease pathogenesis [2]. β-cell-specific Th17 cells accelerate diabetes in mice only after differentiating to a Th1 cell-like phenotype implicating Th17.1 cells in pathogenesis [6, 7].

The development of Th1 and Th17 cells is linked as their differentiation is under control of related heterodimeric cytokines (IL-12 and IL-23, respectively) that share the IL-12 p40 subunit. Thus, p40 blockade should dampen the differentiation of Th1, Th17, and Th17.1 cells. IL-23, and related cytokine pathways, are upregulated in the islets of individuals with T1D [8]. Treatment of NOD mice with a neutralizing antibody to the IL-12 and IL-23 p40 subunit suppresses insulitis and prevents diabetes [9]. T1D is a multifactorial disease and large-scale genome-wide association studies (GWAS) in T1D have identified novel susceptibility loci. Human leukocyte antigen genes (coding for major histocompatibility complex) have the largest effect size. Other susceptibility loci such as CD25 have led to the targeting of Tregs in T1D clinical trials. Interestingly recent GWAS studies have identified novel pathways for clinical targeting including IL-23 [10]. *Ustekinumab* is an antibody with this specificity used to treat psoriasis and inflammatory bowel disease in adults and children [11, 12]. We hypothesized that inhibition of the IL-17A and IFN-γ axes through p40 blockade would delay β-cell destruction.

We present the results of a pilot open-label dose-finding study (NCT02117765) to assess the safety and biologic activity of ustekinumab in the context of T1D. Our independent data and safety monitoring board (DSMB) found no safety concerns at doses used for psoriasis that would prohibit the use of ustekinumab in larger scale placebo-controlled clinical trials. We noted a dose-dependent improvement in biochemical parameters of T1D including suppression of putatively pathogenic Th17.1 immune cells and reduction of proinsulin-specific IFN-γ and IL-17A-producing T cells that have previously been implicated in the pathogenesis of T1D.

## Methods

### Study design and participants

**Figure 1:**
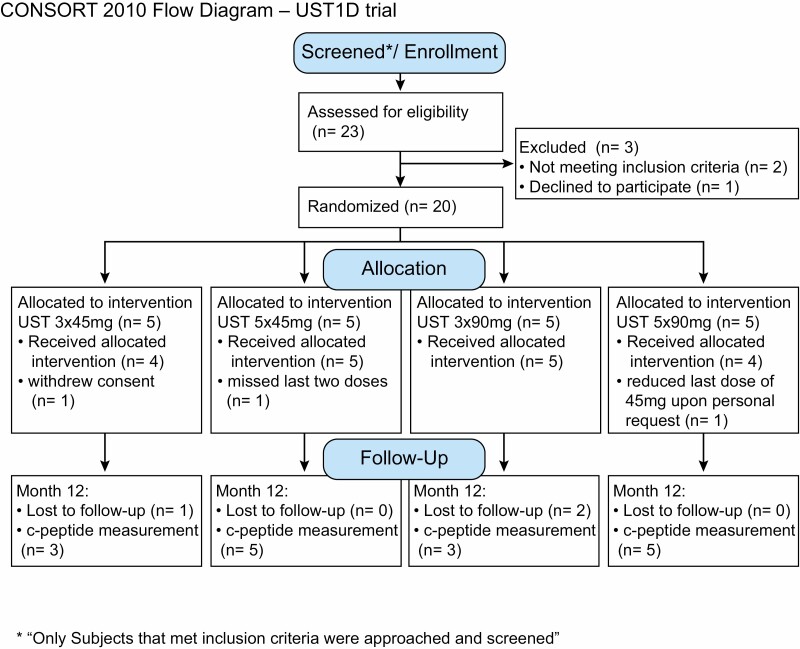
CONSORT Diagram showing allocation and disposition of study subjects adapted from Pesenacker et al., 2019 [13].

Participants in a single-center study were recruited in four sequentially enrolled cohorts receiving either 45 or 90 mg of subcutaneous (SC) ustekinumab for three or five doses over a 1-year period (five participants per group × four groups). These cohorts were: (A) 45 mg-‘maintenance’ SC at weeks 0, 4, 16, 28, and 40 (45 mg × 5); (B) 90 mg-‘maintenance’ SC at weeks 0, 4, 16, 28, and 40 (90 mg × 5); (C) 45 mg-‘loading’ SC at weeks 0, 4, and 16 (45 mg × 3); and (D) 90 mg-‘loading’ SC at weeks 0, 4, and 16 (90 mg × 3) (Fig. 1).

The study was approved by the Research Ethics Board of UBC (H14-00939) and Health Canada. The study was performed in compliance with the guideline for Good Clinical Practice (GCP) of the International Conference of Harmonisation (ICH). Participants eligible to enter the study were aged 18–35 years at enrollment and had a diagnosis of T1D in accordance with ADA/CDA criteria. We included participants within 100 days of diagnosis. Participants were required to have objective evidence of residual functioning β cells defined as a C-peptide level over 0.2 pmol/ml (0.6 µg/l) during a 2-hour Mixed Meal Tolerance Test (MMTT) that was initiated when blood glucose level was 3.9–11.1mmol/l. To objectively differentiate between a T1D and T2D phenotype, participants were required to have a positive test for at least one diabetes-related autoantibody (Glutamate decarboxylase [GAD-65]; IA-2; ZnT8 and/or Insulin, if obtained within 10 days of the onset of exogenous insulin therapy).

Exclusion criteria included the following: anaphylaxis to any human monoclonal antibody; history of malignancy; positive laboratory testing indicating previous or active infection by tuberculosis (purified protein derivative), HIV, hepatitis B or hepatitis C; use of medications known to influence glucose tolerance or immunity; hematological abnormalities; pregnancy; live vaccines 6 weeks prior to the first dose; or major surgery 30 days prior to the first dose.

### Procedures

Ustekinumab (Stelara^®^, Janssen) was purchased from McKesson Canada or supplied by the drug manufacturer and administered to participants at one trial site (BC Diabetes). Pre-filled 45 mg syringes were received, stored and administered to participants at the clinical trial site (BC Diabetes). Subcutaneous injections were administered at weeks 0, 4, 16, 28, and 40 for ‘maintenance dosing’ groups A and B and at weeks 0, 4, and 16 for ‘loading dosing’ groups C and D. A follow-up visit was arranged at 1 year for all participants.

MMTT was performed at the screening visit and visits at weeks 4, 28, and 1 year. Blood was taken at all visits for assessment of safety, diabetes control, and immunological phenotype. Routine hematological and biochemical laboratory assessments were performed at weeks 0, 28, and 1 year.

The primary endpoint was safety as determined by an independent DSMB. This was assessed by the rate, frequency, and severity of all adverse events including hypoglycemic episodes, injection reactions, hypersensitivity reactions, evidence of infection, and posterior leukoencephalopathy syndrome. In addition, adverse events were recorded for any significant deviations in vital signs, standard hematology, and chemistry tests and physical examination.

The pre-specified exploratory endpoints included: MMTT – stimulated 2-hour C-peptide area under the curve (AUC) at weeks 4, 28, and 1 year; [14] insulin use (IU/kg/day) at weeks 4, 16, 28, 40, and 1 year; and HbA1c levels at weeks 4, 16, 28, 40, and 1 year. Secondary immunological mechanistic endpoints were: immune phenotyping via flow cytometry of lymphocytes (including those producing IL-17A and/or IFN-γ); quantification of IL-17A and IFN-γ secretion by Fluorospot.

### Statistical analysis

As this was a phase 1b study, the study was not sized with specific power criteria to determine efficacy. The sample size of 20 provided a 90% probability of detecting at least one adverse event if the rates were as high as 10–11%. Safety was assessed by an independent DSMB. Exploratory endpoints were designed to examine the effect on β-cell function in T1D for different dosing cohorts and immunological mechanistic studies were designed to identify possible dose-dependent biomarkers. These analyses involved comparing baseline with follow-up values using descriptive statistics. Statistical analyses were performed using Prism v7 (GraphPad Software Inc.). Asterisks were used to denote significance; *P < 0.05, **P < 0.01, ***P < 0.001.

### Flow cytometry

Flow cytometry analyses were performed at three independent sites. An interim analysis was performed at the University of British Columbia (UBC) after all participants had reached week 16. This interim analysis focused on CD4^+^ IL-17A and IFN-γ-producing cell subsets (Th17, Th1, and Th17.1). The interim cytometry analysis (conducted at UBC) detected changes in Th17, Th1, Th17.1 cell subsets by staining cryopreserved PBMCs with a combination of antibodies comprising anti-CD3 and anti-CD4 and then stimulated with PMA (100 ng/ml), ionomycin (1 µg/ml) and Brefeldin A (10 µg/ml) for 4 hours before intracellular staining with anti-IL-17A and anti-IFN-γ. Data were acquired on an LSRII cytometer (BD).

A final flow cytometry analysis at the conclusion of the trial included samples from all timepoints and assessed CD4^+^, CD8^+^, and Tregs. This analysis was performed at the T1DUK Immunotherapy Consortium Mechanistic Centre (King’s College London) and the Immune Tolerance Network (ITN) Core Facility (Benaroya Research Institute, Seattle), using flow cytometry platforms that have been validated for use in other T1D clinical trial consortia and in compliance with GCP guidelines. Analysis was performed blinded to treatment group and then analyzed when a final locked data set was sent to the principal investigators.

For the final cytometry analysis (conducted by T1DUK), cryopreserved PBMCs were thawed using RPMI supplemented with 10% FBS (Gibco), spun and resuspended in serum-free X-Vivo 15 (LONZA). Up to 4 million PBMCs were stimulated for 5 hours in 37°C incubator with Leukocyte activation cocktail containing BD GolgiPlug (BD Biosciences) in X-Vivo 15 with 0.5× Monensin (BioLegend). Unstimulated PBMCs were incubated in X-Vivo 15 with 0.5× Monensin and 0.5× Brefeldin (BioLegend). After incubation, cells were stained on ice with Live/Dead fixable near-IR (Thermo Fisher) for 30 minutes. Cells were then incubated with Human TruStain FcX (BioLegend) for 10 minutes before performing surface staining on ice for 30 minutes using a combination of surface stain antibodies. Intracellular staining was performing using FOXP3/transcription factor staining buffer set (Thermo Fisher). Surface marker-stained cells were fixed for 40 minutes at ambient temperature, intracellular stained for 40 minutes at ambient temperature using a combination of intracellular stain antibodies, before being resuspended in FACS buffer for acquisition on BD Symphony flow cytometer and FACSDiva software (BD Biosciences). All samples from the same subject were run on the same day including an internal control sample. Flow data were analyzed using FlowJO Mac version 10 (LCC, BD).

For the confirmatory analysis completed by ITN, cryopreserved PBMCs from all subjects were thawed, incubated with FcX Block and stained with surface antibodies, followed by FOXP3 fix/perm and staining with anti-FOXP3. Instrument standardization was performed using 8 peak rainbow calibration beads (Spherotech, Lake Forest, IL) adjusting PMT voltages for consistent seventh peak mean fluorescent intensities. All samples from the same subject were run on the same day, and an internal control sample from the same subject was run each week. An average of 500,000 live lymphocyte events were collected per sample on a BD Fortessa using Diva software and data was analyzed using FlowJo Mac Version 9.4 (Tree Star Inc., Ashland, OR).

### Fluorospot analysis

Two million cryopreserved PBMCs were stimulated with recombinant GAD65 (Diamyd Medical) [15] and proinsulin (in-kind contribution from Luciano Vilela; Biomm, Brazil) for 46–48 hours. Infanrix, a pentavalent vaccine on the national vaccine schedule for Canada, consisting of purified diphtheria toxoid, purified tetanus toxoid, the acellular component of pertussis vaccine, inactivated poliovirus, and *Haemophilus influenza*e type b polysaccharide was used at 1 µL/mL as a positive control. Following stimulation, non-adherent PBMCs were transferred to pre-coated Fluorospot plates for 18–22 hours. IFN-γ and IL-17A-secreting cells were detected according to manufacturer’s instructions (Mabtech). Enumeration of spots was carried out using the AID Spectrum reader.

### Quantification of serum cytokines

Samples were tested randomized and blinded in duplicates using a next generation electrochemiluminescence based immunoassay format developed by Meso Scale Diagnostics, LLC. Limits of blank (LOB) and lower limit of quantitation of the six assays were as follows: IL-2: 4 fg/ml; 13 fg/ml; IL-10: 9 fg/ml; 80 fg/mL; IL-17A: 100 fg/ml; 200 fg/ml; IL-21: 20 fg/ml; 140 fg/ml; IL-22: 2 fg/ml; 20 fg/ml; IFNg: 30 fg/ml; 90 fg/ml. Dynamic range of each assay was three to four orders of magnitude. Assays were run in a 96-well plate format and read on a SECTOR^®^ Imager. Each plate included eight calibrator levels and four QC samples spanning the measurement range. Analyte concentrations were calculated using a weighted 4PL fit. Calculated concentrations below the LOB were assigned the LOB. Most QC samples recovered within ± 20% of the expected concentrations.

## Results

A total of 20 participants were enrolled, five in each of four dosing cohorts. Protocol-required follow-up was completed by all 10 participants in the two five-dose (maintenance) cohorts, but four participants in the three-dose (induction) groups had loss of follow-up after the third dose at week 16 (Fig. 1). This was because these participants were not due to receive any more doses of drug after week 16 in the induction cohort and they did not want to remain in the study for monitoring. Two participants deviated from the protocol dosing scheme. One participant chose not to take the drug at weeks 28 and 40 and therefore received three doses instead of the planned five doses. One participant requested to have a 45 mg dose at week 40 instead of 90 mg. All other doses were given within the protocol-defined ±7-day window except one participant, who received their 16-week dose 12 days early; the participant was unable to attend on the scheduled day and study staff chose to bring the participant in early rather than remove them from the study. A limitation of this study was the small sample size and the loss to follow up that occurred.

### Study demographics and baseline characteristics

Of the 20 enrolled participants, 12 were female, the mean age was 24.3 (median 22) and ranged from 18 to 35 (Supplementary Table 1). The mean time from diagnosis was 63 days (median 67.5) and ranged from 13 to 98 days. Among all participants, the mean baseline HbA1c was 8.6% (median 8.7) and ranged from 5.8% to 12.2%. The mean baseline weight was 64.5 kg (median 63.9 kg) ranging from 44 to 86 kg. The mean baseline daily insulin use (average of preceding 7 days) was 0.42 IU/kg (median 0.35 IU/kg) and ranged from 0.08 to 1.36 IU/kg.

### Safety analysis

There were 14 adverse events reported, which were evenly balanced between all dosing cohorts (**Table 1**). There were three reported serious adverse events (SAEs). Two reported SAEs occurred in the same participant. The first event – gastroparesis – is a known complication of T1D. The second event was hospitalization due to acute back pain, unrelated to ustekinumab treatment in the opinion of the investigators. The participant requested the final two doses of ustekinumab be withheld; the pain failed to resolve after stopping the drug. The third SAE was due to fatal hypoglycemia. Investigators felt that the hypoglycemia was related to poor diabetes control. The participant was not compliant with taking insulin as prescribed and monitoring their blood glucose levels during a mountaineering expedition. In addition, there was absence of medical management to detect the hypoglycemic event. The participant’s last ustekinumab dose was more than 12 weeks prior to the incident, thus there would not have been active drug in the participant’s body at the time of death. Post-marketing data of over 40,000 patient years has not identified any increase in rate of all-cause mortality with ustekinumab therapy [16, 17]. This serious adverse event was deemed unrelated to ustekinumab by our principal clinical investigator and independent DSMB.

**Table 1:** Summary of serious adverse events and non-serious adverse events

Cohort	Age	Sex	Study day of onset	Last dose received	Description	Severity	Intervention required	Outcome	Relationship to ustekinumab
45 mg × 5	23	F	20	Dose 1	Acute diabetic neuropathic pain; treated in hospital	Serious Adverse Event	Required hospitalization	Discharged from hospital	Unrelated
	23	F	118	Dose 3	Hospitalized for acute exacerbation from back pain T5 to low lumbar	Serious Adverse Event	Required hospitalization	Discharged from hospital	Unrelated
	23	F	-4	Prior to Baseline	Influenza	Mild Adverse Event	None	Resolved with no seq	Unrelated
	23	F	78	Dose 2	Urinary tract infection	Mild Adverse Event	Concomitant meds	Resolved with no seq	Unlikely
	31	M	340	Dose 4	Influenza	Mild Adverse Event	None	Resolved with no seq	Unrelated
	20	F	20	Dose 1	Influenza	Mild Adverse Event	Concomitant meds	Resolved with no seq	Unrelated
90 mg × 5	28	F	13	Dose 1	Bacterial Vaginosis	Severe Adverse Event	Concomitant meds (metronidazole from June 22-June 29)	Resolved with no seq	Possibly
	35	M	24	Dose 2	Hallucinations 2–3 weeks after dosing. Started after second dose, lasting 1 month	Moderate Adverse Event	None	Resolved with no seq	Possibly
	35	M	100	Dose 2	Upper respiratory tract infection	Mild Adverse Event	None	Resolved with no seq	Unrelated
45 mg × 3	18	M	174	Dose 3	Upper respiratory tract infection	Mild Adverse Event	Non-drug therapy	Resolved with no seq	Unrelated
90 mg × 3	30	F	260	Dose 3	Death	Serious Adverse Event	N/A	Death	Unrelated
	30	F	89	Dose 2	Upper respiratory tract infection	Mild Adverse Event	Non-drug therapy	Resolved with no seq	Unrelated
	27	F	64	Dose 2	Fracture of right wrist	Moderate Adverse Event	Other	Resolved with no seq	Unrelated

Three serious adverse events were not thought to be related to ustekinumab administration. Ten non-serious adverse events occurred during the trial. Most AEs (7/10) were mild infections that resolved with no sequelae. Two (hallucinations and wrist fracture) were moderate in severity but not thought to be related to study drug. One adverse event was a severe episode of bacterial vaginosis that was possibly related to study drug administration.

Although hallucinations and paranoia reported by one participant (90 mg × 5) did not immediately impact ustekinumab dosing, the participant requested to have his/her third dose reduced. This adverse event was therefore ascribed to be potentially related to ustekinumab. One event was considered severe at presentation (90 mg × 5 cohort; bacterial vaginosis), but with appropriate treatment, it resolved with no sequelae within 7 days of onset. Several of the adverse events related to minor infections. Six participants had minor upper respiratory tract infections. These resolved without the need for therapy (beyond over the counter standard comfort medications) and were in keeping with usual rates seen in a young adult population. One participant acquired a urinary tract infection that resolved with standard treatment and without sequelae. This was not thought to be related to the study drug. There were no other major hypoglycemic episodes and no injection reactions. There were no major deviations in hematological and biochemical laboratory parameters. The final report of the independent DSMB found no safety concerns that would prohibit the use of ustekinumab in larger scale placebo-controlled clinical trials in T1D.

### Mechanistic analysis

**Figure 2:**
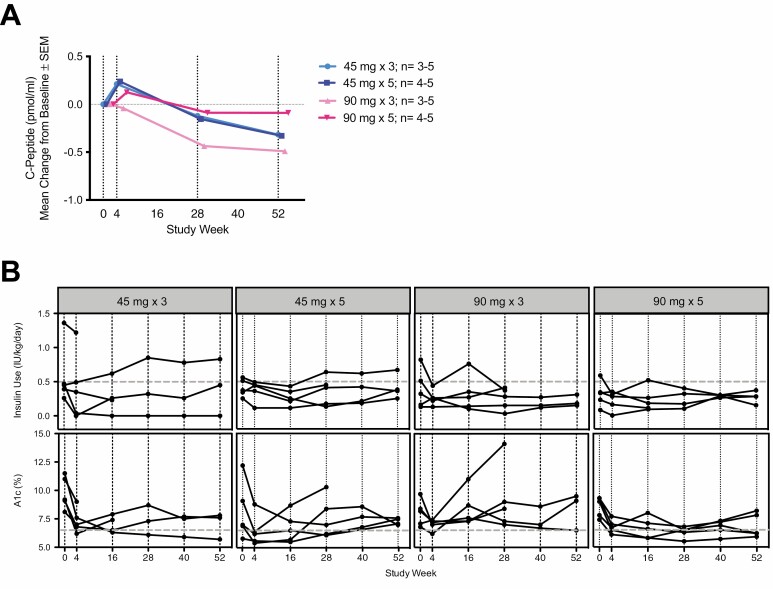
Summary of clinical endpoint data in young adults with new onset T1D receiving ustekinumab. (A) The mean change from baseline of C-peptide MMTT AUC over time was compared between subjects treated with a maintenance dose (five-dose regimen) of 45 mg or 90 mg (navy and red, respectively; week 52 *P* = 0.081) and between subjects receiving a 90 mg loading dosing (three doses) or maintenance dosing (five doses) (pink and red, respectively; week 52 *P* = 0.246). (B) Insulin dose and glycated hemoglobin over time for all subjects (intention-to-treat). Dotted lines indicate the cut off for the pre-defined clinical responder status. Data from subjects 26632 and 26491 were excluded after weeks 16 and 28, respectively, due to protocol deviation.

A limitation of the study is that, given the small sample size, the cohorts had imbalances that could have affected the mechanistic analysis. A decline in C-peptide MMTT AUC is typical during the first year following diagnosis of T1D (approximately 0.3 pmol/ml/year) [18], and was least in the participants receiving the highest drug dose (Fig. 2A). Most participants maintained concurrent HbA1c ≤ 6.5% and insulin use ≤ 0.5 IU/kg/day (Fig. 2B).

**Figure 3:**
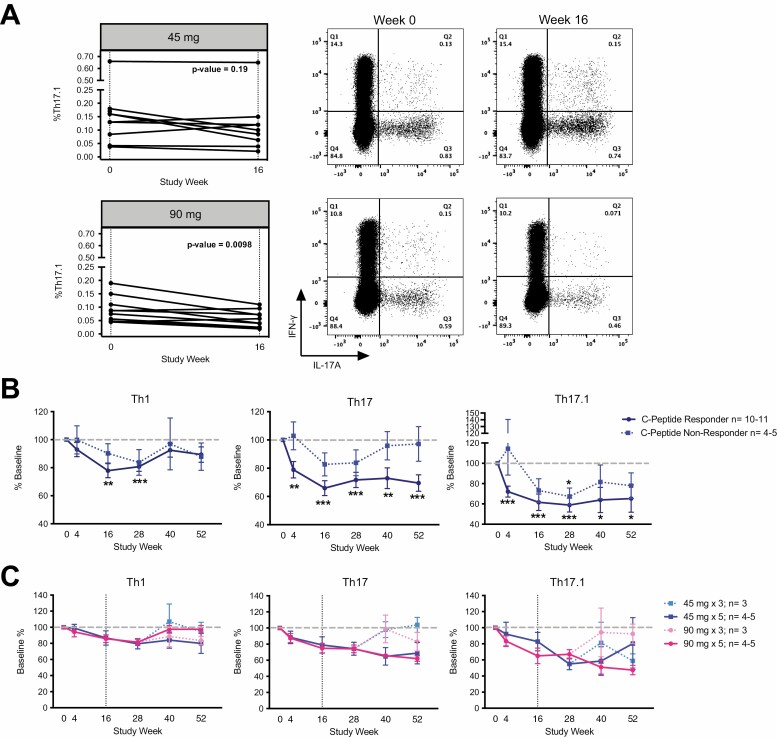
Ustekinumab at 90 mg dose reduces the proportion of Th17.1 cells. (A) Flow cytometry analyses of cryopreserved PBMCs revealed that subjects treated with the 90 mg dose of ustekinumab had a significant reduction in the proportion of IL-17A^+^IFN-γ ^+^CD4^+^ Th17.1 cells at week 16 (Wilcoxon test, *n* = 9–10). Representative dot plots from weeks 0 and 16 are shown. (B) Flow cytometry analyses of cryopreserved PBMCs revealed that reduction in CD4+IL-17A+ T cells (Th17) and IL-17A^+^IFN-γ ^+^CD4^+^ cells (Th17.1) following therapy was more pronounced in subjects defined as C-peptide responders (individuals with a loss of C peptide of less than 0.3 pmo/ml/year) compared to C-peptide non-responders and this reached significance at all weeks. (mixed-effects model with Geisser-Greenhouse correction, individual *P*-values corrected using Original FDR method of Benjamini and Hochberg; * *P* ≤ 0.05; ** *P* ≤ 0.01; *** *P* ≤ 0.001.). (C) Reduction of cytokine production upon ustekinumab administration reverted to baseline when drug administration was stopped at weeks 48 and 52 in both 45 mg and 90 mg three-dose (induction; dashed) groups. Vertical dashed gray line = discontinued ustekinumab (induction group). Subjects 26632 and 26491 excluded after weeks 16 and 28, respectively, where applicable due to protocol deviation. Horizontal dashed grey line = Baseline (100%).

During an interim analysis, when all enrolled participants had reached week 16 (loading drug dose), the frequency of circulating Th17.1 (dual producing IL-17A and IFN-γ CD4^+^ T cells) in the low (45 mg) and high (90 mg) dosing cohorts (*n* = 10) was assessed at UBC. The proportion of Th17.1 cells was significantly reduced from baseline (screening visit sample) to week 16 in the 90 mg (*P* = 0.037) but not in the 45 mg (*P* = 0.65) dosing group (Supplementary Fig. 1A). Following trial completion, a final immune-phenotype analysis of all cohorts and timepoints by the T1DUK Immunotherapy Consortium Mechanistic Centre independently confirmed the finding that the proportion of Th17.1 cells was reduced from week 0 to week 16 in the 90 mg (*P* = 0.0098), but not the 45 mg group (*P* = 0.19) (Fig. 3A). The absolute percentages of Th17.1 were different between the two analyses due to difference in antibodies and gating strategies. However, the concordance of the two independent analyses on different aliquots of cryopreserved PBMC samples added validity to the finding that higher doses of ustekinumab more effectively reduce the proportion of Th17.1 cells.

C peptide-responder individuals were defined as those with a loss of C peptide of less than 0.3 pmo/ml/year, and were identified in all cohorts. Due to the low numbers of participants in each dosing cohort, we proceed with a pooled analysis that showed C peptide responders had enhanced reduction in frequencies of Th1, Th17, and Th17.1 cells when compared with non-responders, although this drop was only significant in the Th17 and Th17.1 cell populations (Fig. 3B). Ustekinumab administration was found to reduce the proportions of Th1, Th17, and Th17.1 cells, as defined by cytokine production, at all time-points, with resolution (normalization/return to baseline) after treatment cessation (Fig. 3C). Thus, ustekinumab inhibited proinflammatory CD4^+^ T cells in adults with T1D. A third set of flow cytometry data, generated by the ITN at the Benaroya Research Institute, further corroborated the results obtained at the UBC and UK sites demonstrating the robustness of these observations (Supplementary Fig. 1B and C).

We also measured drug levels in a proportion of subjects and found that at week 16 drug levels peaked and at week 28 drug levels reached a steady state (data not shown). At week 4, 16, 28, 40, and week 52, when all subjects were pooled, we observed reductions in IL-22 (a Th17-related cytokine), but no sustained changes in the proportions of Tc17 or Treg cells (Supplementary Fig. 2). Changes in T cell subsets were mirrored by changes in serum cytokine profiles, although data were only available for early timepoints in the study for this analysis (Supplementary Fig. 3).

**Figure 4:**
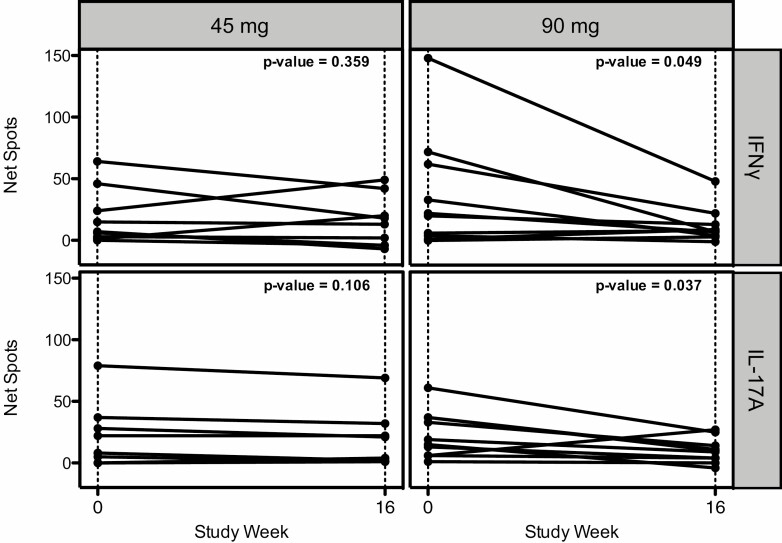
The 90 mg but not 45 mg, ustekinumab dose reduces the proportion of proinsulin-specific IL-17A and IFN-γ-producing CD4^+^ T cells in young adults with new onset T1D at week 16. Fluorospot analysis was used to count the proinsulin-specific CD4^+^ T cells by subtracting proinsulin-stimulated PBMCs from control PBS-stimulated cells to ascertain net spots. There was a significant reduction in proinsulin-specific IFN-γ and IL-17A-producing CD4^+^ T cells from week 0 to week 16 in the 90 mg dosing cohorts (Wilcoxon test, *n* = 5).

We assessed if ustekinumab affected islet-specific immune responses to proinsulin and GAD65, which have been proposed as recognition targets in T1D [19]. Using Fluorospot, we found a significant decrease between week 0 and week 16 in proinsulin-specific IFN-γ and IL-17A T cell responses in the 90 mg cohort (IL-17A, *P* = 0.037; IFN-γ, *P* = 0.049; Fig. 4). In contrast, there were no changes in the proportion of the GAD65-specific cells that produced IFN-γ and/or IL-17A (Supplementary Table 2). Decreased IL-17A responses to control antigens but not to IFN-γ were noted in the higher dose cohort.

## Discussion

To our knowledge, this is the first reported study of ustekinumab in individuals with T1D. Ustekinumab is approved therapy for psoriasis in adults and in children 12 years of age or older [20, 21]. In a psoriasis population, post-marketing data of over 40,000 patient years has not identified any increase rate of malignancy, serious infection, or all-cause mortality [16, 17]. The serious adverse events described in this study have not been previously noted and instead were determined by the principal investigators and DSMB to be complications of poor T1D control. The death that occurred in the study was in a participant that had a dose of ustekinumab more than 12 weeks prior, indicating the drug would no longer be active. There were extenuating factors (poor compliance with treatment and extreme strenuous activity without access to medical care) to explain the death of this participant. These factors likely contributed to the independent DSMB agreeing that the death was not secondary to ustekinumab and determining that the drug was safe to progress to further efficacy studies. In summary, this study demonstrates that ustekinumab given to the adult T1D population at a maximum dose of 90 mg at baseline, at 1 month (loading dose), and then every 12 weeks (maintenance) did not result in any serious adverse events attributed to the drug. Adverse events recorded in the study related to the study drug were mild, transient, and did not increase in frequency as the ustekinumab dose was increased between the cohorts.

The descriptive analysis of the exploratory endpoints indicated that the highest dose of ustekinumab (90 mg × 5) resulted in the smallest decline in C-peptide AUC in response to MMTT at 1 year. However, the baseline C-peptide MMTT AUC was higher in this group than in the other dosing cohorts. We did not fully measure all the covariates of participants that could impact this main outcome measure such as genetic risk contribution or EBV/CMV serostatus. Genetic risk susceptibility loci (e.g. IL-23A polymorphisms) distribution between subjects could explain the presence of C-peptide responders versus non-responders for ustekinumab [10]. Although this study was not powered to show statistical significance between dosing groups, these data suggest that a higher induction and maintenance dosing regimen of ustekinumab, as used in Crohn’s disease [22], may be more effective in meeting clinical efficacy endpoints for future studies.

In psoriasis, the keratinocyte proliferation that leads to skin lesions is a response to increased IL-17A production by memory Tc17 cells under the control of IL-23, providing an explanation for the efficacy of ustekinumab in this disease [23]. Th17.1 cells and Th1 cells are similarly implicated in the immune-pathogenesis of T1D, and this study aids in our understanding of how ustekinumab impacts these cell types in peripheral blood of individuals with T1D. Participants in the 90 mg maintenance cohort showed a reduction in circulating Th17.1 cells, defined by co-production of IFN-γ and IL-17A, and proinsulin-specific IFN-γ and IL-17A-producing T cells, suggesting that these parameters could serve as biomarkers for efficacy. The robustness of these observations is underscored by the concordant results obtained by immunophenotyping of cohort samples at three independent sites. To our knowledge, these data are the first to demonstrate specific inhibition of putative pathogenic Th1/17/17.1 cells in human T1D without attendant broad immunosuppression. Limitations to this conclusion are that there is also a general decrease in IL-17 secreting cells with ustekinumab (as noted in responses to control antigens by Flurospot). We suggest that higher doses of ustekinumab are likely to produce the most favorable outcomes in terms of improving disease related biochemical parameters.

Targeted immune modulation of T1D has been a primary goal of recent studies in individuals with early and incipient disease but has been hampered by concerns over immunosuppression [24]. Teplizumab, an anti-CD3 antibody targeting all T cells, has shown efficacy in preventing T1D progression in relatives at risk for T1D [25]. The teplizumab studies established the principle that T cell directed therapy over a short period of two weeks is efficacious in T1D and has an acceptable safety profile [24, 26]. Ustekinumab is a more targeted treatment with a good safety profile in other diseases, which may reduce hesitancy to treat pre-symptomatic individuals with immunosuppression. Unlike teplizumab, ustekinumab therapy for psoriasis and inflammatory bowel disease requires ongoing administration, potentially decreasing enthusiasm. It remains to be determined if early use decreases the need for chronic administration. The targeting of other pro-inflammatory pathways such as IL-21 and tumor necrosis factor (TNF) in T1D may also require chronic therapy [27, 28]. Alternative strategies are to enhance regulatory immune responses with Treg cell infusions or IL-2 or to induce antigen-specific tolerance [29, 30]. A combination of timed strategies, blocking effector mechanisms, and enhancing tolerance may eventually prove optimal. We suggest that a placebo-controlled efficacy clinical trial of ustekinumab is warranted in individuals with early onset T1D. This might provide a rationale for potential therapeutic utility in T1D of more potent inhibitors of the IL-23 pathway, in current or planned use in psoriasis and inflammatory bowel disease, such as guselkumab, risankizumab, or TYK2 inhibition [31].

## Supplementary material

Supplementary data are available at *Immunotherapy Advances* online.

Supplementary Figure 1: (A) Flow cytometry analyses conduct at UBC of cryopreserved PBMCs revealed that subjects treated with the 90mg dose of ustekinumab had a significant reduction in the proportion of IL-17A^+^IFN-γ ^+^CD4^+^ Th17.1 cells at week 16 (Wilcoxon test, n=9-10). **(B)** Heatmap showing percent change from baseline (week 0) to week 16 of specified T cell populations. P-values for percent change are displayed in a heatmap for naïve, effector memory, central memory, effector memory RA, or Tr1 phenotypes in CD4^+^ conventional T cells or CD8^+^ T cells. Ustekinumab does not alter relative frequencies of any of these cell populations. Data were generated at the Benaroya institute by the Immune Tolerance Network (ITN) using cryopreserved PBMCs. **(C)** Correlation plots comparing Th17.1 flow cytometry data obtained from three sites. Correlation plots of week 0 and 16 flow cytometry data (shown as a proportion of CD4^+^ T cells) obtained by running replicate aliquots of cryopreserved PBMCs at three different centers: UBC (interim analysis), UK (final analysis) and ITN (validation analysis). Th17.1 cell enumeration was consistent between all three analyses with Spearman’s Rho ranging between 0.53-0.69 and p-values <0.0001.

Supplementary Figure 2: **(A)** Longitudinal immunophenotyping analysis showed a sustained effect of ustekinumab administration, with significantly reduced proportions of Th17.1, Th1 and Th17 cells detected until week 52 (Wilcoxon test, n=16-20, *p<0.05, **p<0.01, ***p<0.001). Proportions of other Th17 cell-associated cytokines (IL-21, IL-22, GM-CSF, IL-10), Tc17 and Treg cells are shown for comparison. **(B)** IL-22 and GM-CSF flow cytometry analyses of cryopreserved PBMCs in subjects defined as C-peptide responders (individuals with a loss of C peptide of less than 0.3pmo/ml/year) compared to C-peptide non-responders (Mixed-effects model with Geisser-Greenhouse correction, individual p-values corrected using Original FDR method of Benjamini and Hochberg; * p ≤ 0.05; ** p ≤ 0.01; *** p ≤ 0.001.) Subjects 26632 and 26491 excluded after weeks 16 and 28, respectively, for panel B.

Supplementary Figure 3: Consistent with its mechanism of action, serum levels of Th17/17.1 cytokines, IL-17A, IL-10, IFN-γ, IL-2, IL-21, and IL-22, were reduced following ustekinumab treatment at week 4 and 16. * p ≤ 0.05

Supplementary Table 1: Summary of cohort demographics and baseline characteristics are shown. Baseline age, sex, days from diagnosis to first dose, weight, insulin use, HbA1c and 2-Hour MMTT C-peptide in four dosing cohorts (45mg x 3), (45mg x 5), (90mg x 3) and (90mg x 5). The top row shows mean (SD) or N (%) and bottom row shows Median [Min, Max].

Supplementary Table 2: Fluorospot data p-values (Wilcoxon test) differences between week 16 and week 0 in IFN-γ and IL-17A-secreting T cells stimulated with proinsulin, GAD65, Infanrix/Candida. Table shows all samples combined and 90mg and 45mg cohorts separated. Shaded boxes indicate significance.

ltab022_suppl_Supplementary_Figure_S1

ltab022_suppl_Supplementary_Figure_S2

ltab022_suppl_Supplementary_Figure_S3

ltab022_suppl_Supplementary_Table_S1

ltab022_suppl_Supplementary_Table_S2

## Data Availability

Clinical data and protocol can be found on ClinicalTrial.gov using the identifier NCT02117765 shortly after the publication of this article. Other data are available upon request from the authors.

## Abbreviations

AUC: Area under the curve
CD: Cluster of differentiation
CMV: Cytomegalovirus
DSMB: Data and safety monitoring board
EBV: Epstein-Barr virus
GAD65: Glutamate decarboxylase 65
GCP: Good clinical practice
GWAS: Genome-wide association studies
HIV: Human immunodeficiency virus
IA-2: Islet Antigen 2
ICH: International Conference of Harmonisation
IFN-γ: Interferon-γ
IL-12: Interleukin-12
IL-17A: Interleukin-17a
IL-23: Interleukin-23
IU: Insulin Units
LOB: Limits of blank
MMTT: Mixed meal tolerance test
NOD: Non-obese diabetic
PBMC: Peripheral blood mononuclear cells
SAE: Serious adverse events
SC: Subcutaneous
T1D: Type 1 diabetes
T2D: Type 2 diabetes
Tc: Cytotoxic T cells
Th: T helper cells
Treg: T regulatory cells
TYK2: Tyrosine kinase 2
ZnT8: Zinc transporter 8
