# Supplementary figures and images for: A phase 1b open-label dose-finding study of ustekinumab in young adults with type 1 diabetes
Source: Immunother Adv. 2021 Nov 13;2(1):ltab022. doi: 10.1093/immadv/ltab022 (PMC8769169; doi:10.1093/immadv/ltab022)

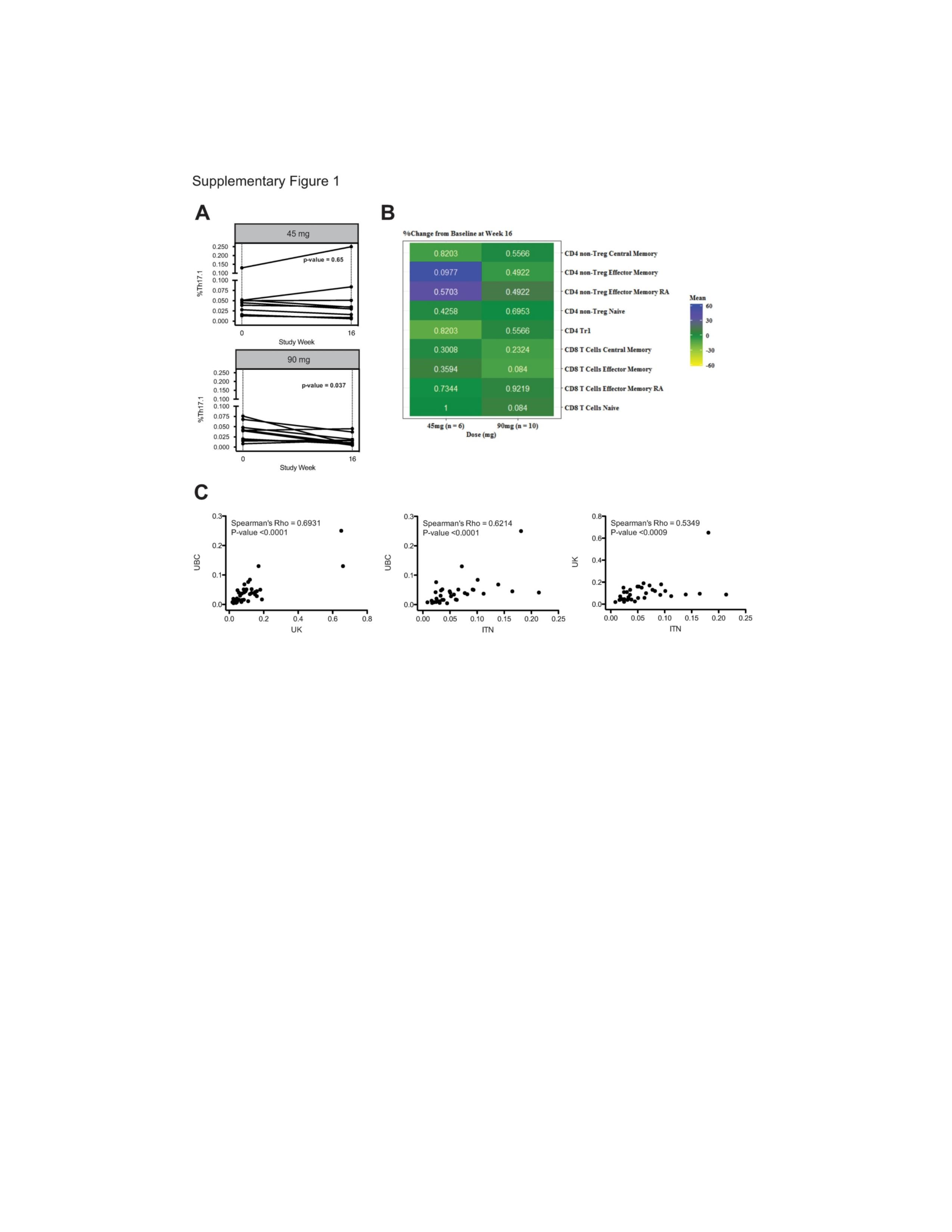

Supplement: ltab022_suppl_Supplementary_Figure_S1 [file ltab022_suppl_supplementary_figure_s1.jpeg]

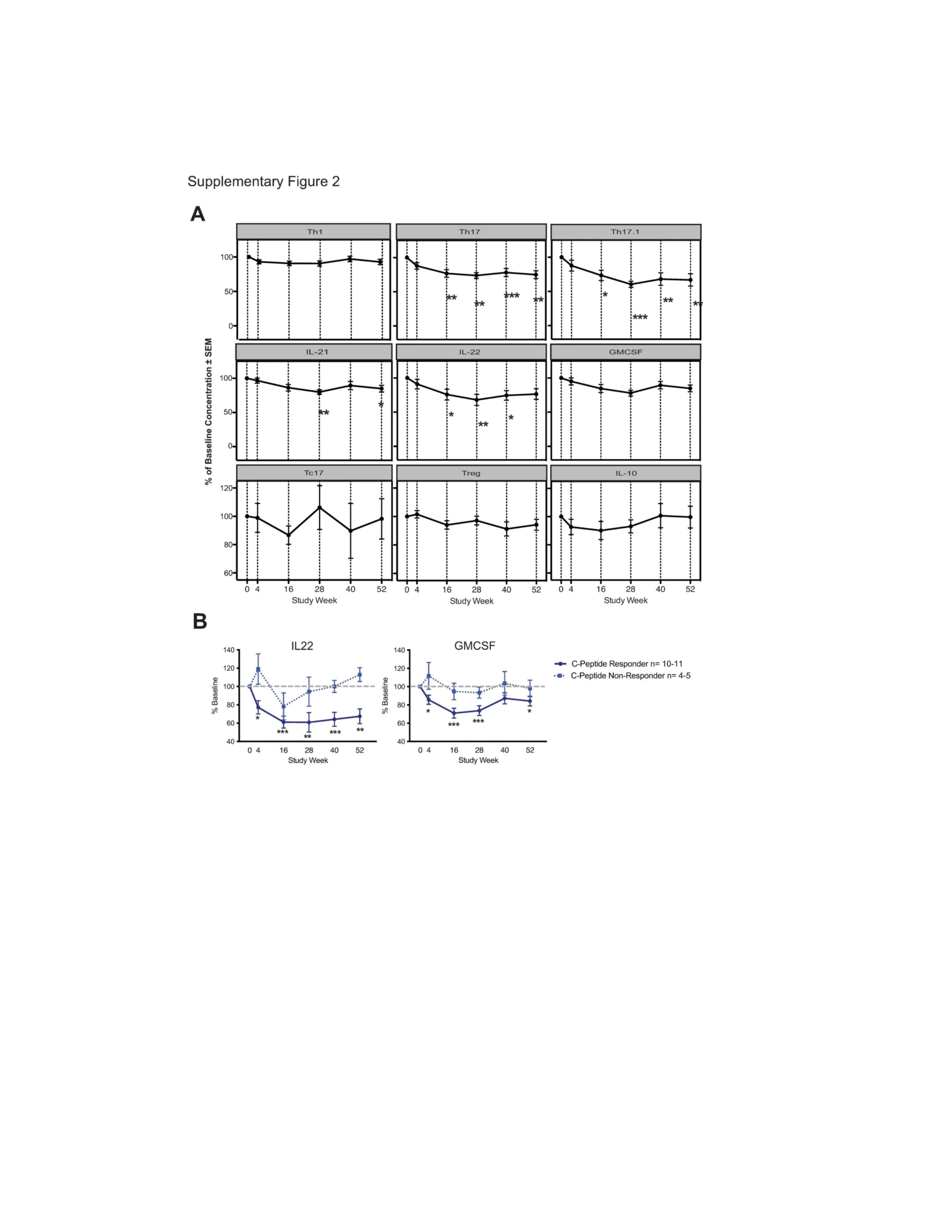

Supplement: ltab022_suppl_Supplementary_Figure_S2 [file ltab022_suppl_supplementary_figure_s2.jpeg]

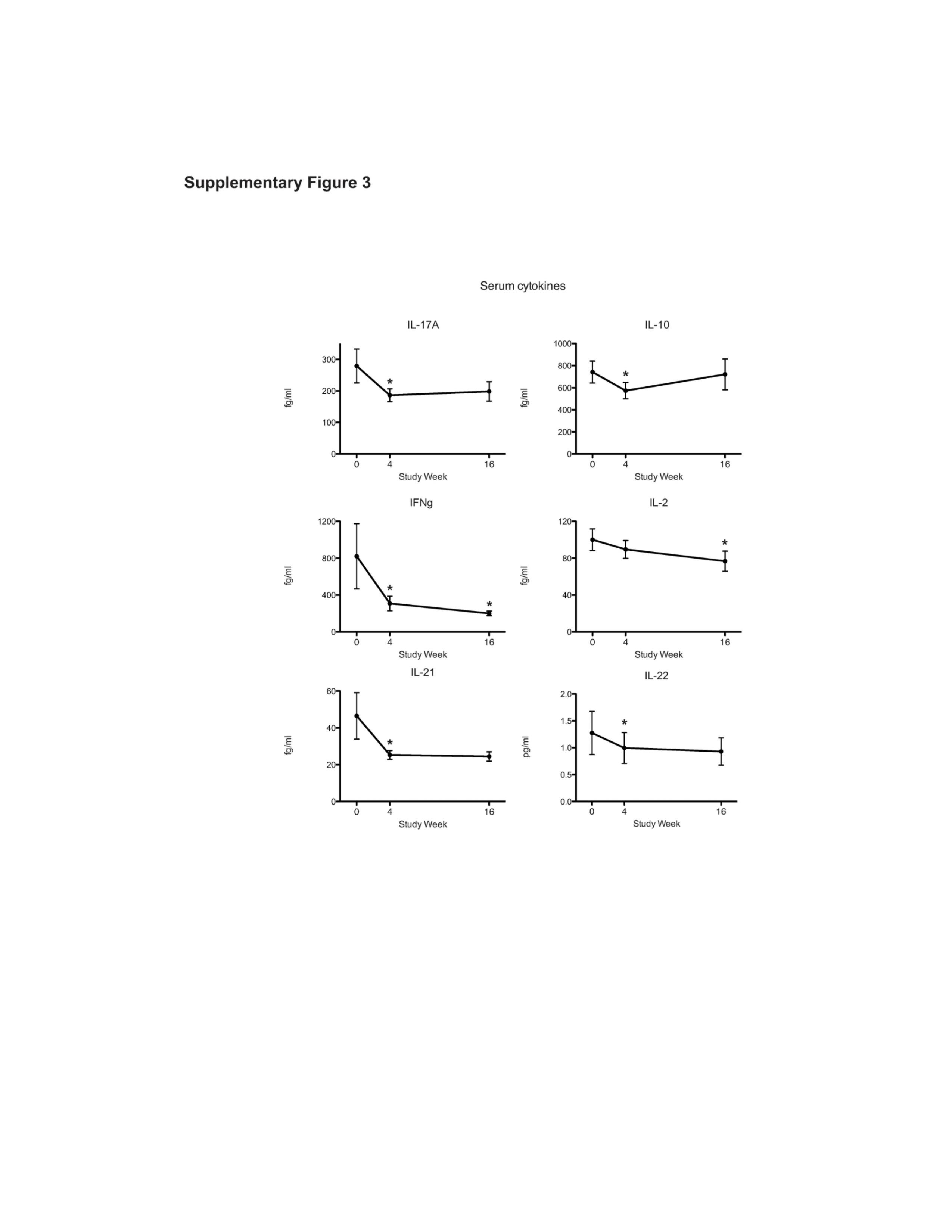

Supplement: ltab022_suppl_Supplementary_Figure_S3 [file ltab022_suppl_supplementary_figure_s3.jpeg]
