# Supplementary material for: A phase 1b open-label dose-finding study of ustekinumab in young adults with type 1 diabetes
Source: Immunother Adv. 2021 Nov 13;2(1):ltab022. doi: 10.1093/immadv/ltab022 (PMC8769169; doi:10.1093/immadv/ltab022)
Supplement: ltab022_suppl_Supplementary_Table_S1 [file ltab022_suppl_supplementary_table_s1.docx]

**Supplementary Table 1:** **Summary of cohort demographics and baseline.**

| **Variable** |  | **45 mg X 3** | **45 mg X 5** | **90 mg X 3** | **90 mg X 5** | **Total** |
| --- | --- | --- | --- | --- | --- | --- |
| **Age   (yrs)** | Mean (SD)  Median [Min, Max] | 24 (7·38) 20 [18, 33] | 22·8 (4·82) 21 [19, 31] | 25·4 (5·27) 27 [18, 30] | 25 (6·52) 22 [19, 35] | 24·3 (5·68) 22 [18, 35] |
| **Sex (Female)** | N (%) | 2 (40%) | 3 (60%) | 4 (80%) | 3 (60%) | 12 (60%) |
| **Days from Dx   to first Dose** | Mean (SD)  Median [Min, Max] | 55·4 (32·8) 50 [13, 98] | 61·6 (20·45) 68 [30, 84] | 68·4 (16·74) 67 [52, 96] | 66·6 (20·95) 68 [32, 86] | 63 (22·2) 67·5 [13, 98] |
| **Weight (kg)** | Mean (SD)  Median [Min, Max] | 62·5 (9·43) 57·1 [54·2, 73·5] | 68·1 (10·53) 67·6 [52·7, 79] | 59·22 (12·24) 60·4 [44, 76·5] | 68·04 (12·94) 65 [55·9, 85·8] | 64·47 (11·13) 63·85 [44, 85·8] |
| **Insulin Use (IU/kg/day)** | Mean (SD)  Median [Min, Max] | 0·59 (0·44) 0·45 [0·26, 1·36] | 0·41 (0·13) 0·37 [0·25, 0·56] | 0·39 (0·28) 0·32 [0·13, 0·82] | 0·31 (0·19) 0·33 [0·08, 0·59] | 0·42 (0·28) 0·35 [0·08, 1·36] |
| **HbA1c (%)** | Mean (SD)  Median [Min, Max] | 9·78 (1·42) 9·2 [8·1, 11·5] | 8·2 (2·53) 7 [5·8, 12·2] | 8·04 (1·15) 8·2 [6·8, 9·7] | 8·56 (0·9) 9 [7·4, 9·3] | 8·64 (1·65) 8·7 [5·8, 12·2] |
| **2-Hour MMTT C-Peptide (pmol/ml)** | Mean (SD)  Median [Min, Max] | 0·53 (0·24) 0·43 [0·25, 0·8] | 0·53 (0·11) 0·56 [0·35, 0·64] | 0·57 (0·24) 0·47 [0·29, 0·83] | 0·79 (0·37) 0·62 [0·46, 1·4] | 0·61 (0·26) 0·57 [0·25, 1·4] |
