# Supplementary material for: A phase 1b open-label dose-finding study of ustekinumab in young adults with type 1 diabetes
Source: Immunother Adv. 2021 Nov 13;2(1):ltab022. doi: 10.1093/immadv/ltab022 (PMC8769169; doi:10.1093/immadv/ltab022)
Supplement: ltab022_suppl_Supplementary_Table_S2 [file ltab022_suppl_supplementary_table_s2.pptx]

## Slide 1
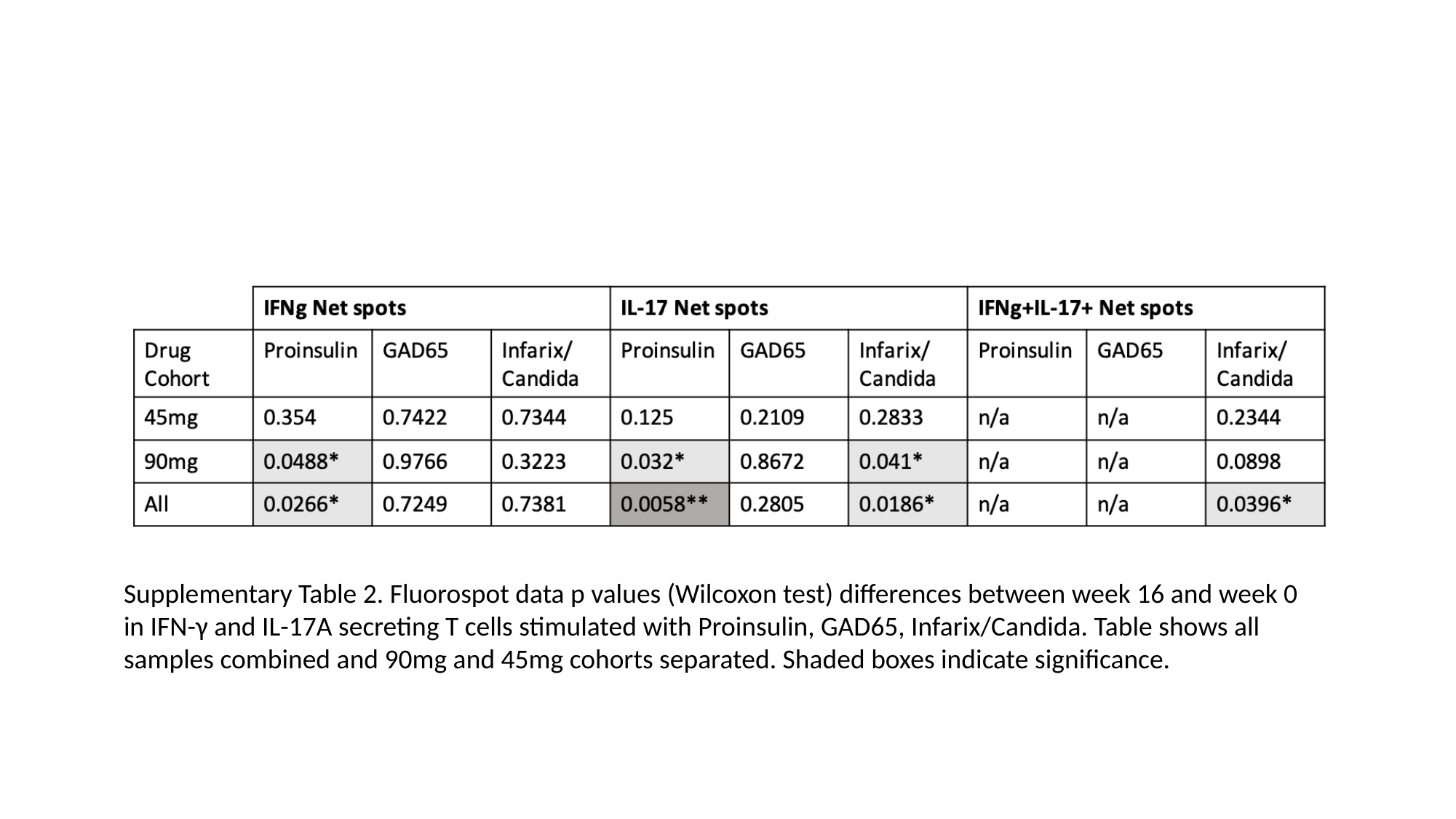

Supplementary Table 2. Fluorospot data p values (Wilcoxon test) differences between week 16 and week 0 in IFN-γ and IL-17A secreting T cells stimulated with Proinsulin, GAD65, Infarix/Candida. Table shows all samples combined and 90mg and 45mg cohorts separated. Shaded boxes indicate significance.
